# 216. Bacterial Genotype and Clinical Outcomes in Solid Organ Transplant Recipients with *Staphylococcus aureus* Bacteremia

**DOI:** 10.1093/ofid/ofab466.418

**Published:** 2021-12-04

**Authors:** Emily Eichenberger, Felicia Ruffin, Batu K Sharma-Kuinkel, Michael M Dagher, Larry Park, Matthew Sinclair, Celia Kohler, Vance G Fowler, Stacey Maskarinec

**Affiliations:** 1 Duke University, Durham, North Carolina; 2 Duke University Medical Center, Durham, North Carolina; 3 Duke University Department of Medicine, Durham, North Carolina

## Abstract

**Background:**

Solid organ transplant (SOT) recipients are characterized by extensive healthcare exposure and have high rates of *Staphylococcus aureus* bacteremia (SAB). The clinical characteristics and outcome determinants of SAB in this population are poorly understood. We undertook a prospective cohort study compared the bacterial genotype and clinical outcomes of SAB among SOT and non-solid organ transplant (non-SOT) recipients.

**Methods:**

Consecutive patients presenting to our institution with SAB between January 1, 2016 and December 31, 2019 were eligible for study inclusion. Each subject’s initial *S. aureus* bloodstream isolate was genotyped using *spa* typing and assigned to clonal complexes using Ridom StaphType software.

**Results:**

A total of 32 SOT and 634 non-SOT recipients with SAB were included. Bacterial genotype did not differ significantly between SOT and non-SOT recipients (p=0.4855), including the proportion of SAB caused by USA300 (12.5% vs 16.7%, p=0.6339). Ninety-day mortality and incidence of metastatic complications did not significantly differ between SOT and non-SOT recipients (18.8% vs 30.1%, p=0.2329, and 37.5% vs 48.6%, p=0.2769, respectively). Transplant status was significantly associated with septic shock (50.0% vs 21.8%, adjusted OR 2.63, 95% CI: 1.22 to 5.66). Infection with USA300 was not associated with 90-day mortality or septic shock among SOT recipients (p=1.0000 for both).

*Staphylococcus aureus* Genotype by Transplant Status

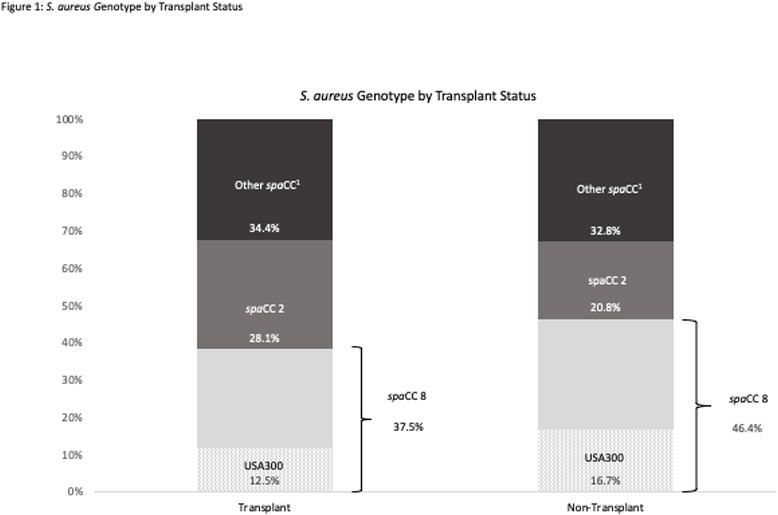

**Conclusion:**

In conclusion, SOT recipients with SAB do not experience greater mortality than non-SOT recipients. Differences in genotype of the infecting bacteria do not appear to be a significant determinant of outcome in SOT recipients with SAB.

**Disclosures:**

**Vance G. Fowler, Jr., MD, MHS**, **Achaogen** (Consultant)**Advanced Liquid Logics** (Grant/Research Support)**Affinergy** (Consultant, Grant/Research Support)**Affinium** (Consultant)**Akagera** (Consultant)**Allergan** (Grant/Research Support)**Amphliphi Biosciences** (Consultant)**Aridis** (Consultant)**Armata** (Consultant)**Basilea** (Consultant, Grant/Research Support)**Bayer** (Consultant)**C3J** (Consultant)**Cerexa** (Consultant, Other Financial or Material Support, Educational fees)**Contrafect** (Consultant, Grant/Research Support)**Debiopharm** (Consultant, Other Financial or Material Support, Educational fees)**Destiny** (Consultant)**Durata** (Consultant, Other Financial or Material Support, educational fees)**Genentech** (Consultant, Grant/Research Support)**Green Cross** (Other Financial or Material Support, Educational fees)**Integrated Biotherapeutics** (Consultant)**Janssen** (Consultant, Grant/Research Support)**Karius** (Grant/Research Support)**Locus** (Grant/Research Support)**Medical Biosurfaces** (Grant/Research Support)**Medicines Co.** (Consultant)**MedImmune** (Consultant, Grant/Research Support)**Merck** (Grant/Research Support)**NIH** (Grant/Research Support)**Novadigm** (Consultant)**Novartis** (Consultant, Grant/Research Support)**Pfizer** (Grant/Research Support)**Regeneron** (Consultant, Grant/Research Support)**sepsis diagnostics** (Other Financial or Material Support, Pending patent for host gene expression signature diagnostic for sepsis.)**Tetraphase** (Consultant)**Theravance** (Consultant, Grant/Research Support, Other Financial or Material Support, Educational fees)**Trius** (Consultant)**UpToDate** (Other Financial or Material Support, Royalties)**Valanbio** (Consultant, Other Financial or Material Support, Stock options)**xBiotech** (Consultant)

